# The mediating role of psychological resilience in the relationship between feelings of alienation and self-care ability among rural elderly living alone: A cross-sectional study

**DOI:** 10.1097/MD.0000000000045053

**Published:** 2025-10-17

**Authors:** Li-Hong Yang, Lingyun Qu

**Affiliations:** a College of Nursing, Chifeng University‌, Chifeng, Inner Mongolia, China; b University of Sanya, Sanya, Hainan, China.

**Keywords:** mediating effect, psychological resilience, rural elderly living alone, self-care ability in old age, sense of alienation

## Abstract

This paper explores the relationship between the self-care capabilities of elderly individuals living alone in rural areas and their feelings of alienation and psychological resilience. The objective is to provide references for enhancing the self-care abilities of these rural elderly individuals living alone. In this study, we employed a general information questionnaire, a Senior Isolation Scale, a Psychological Resilience Scale, and a Self-Elderly Care Ability Scale to survey 925 rural empty-nest elderly residents in Hulunbuir city, Inner Mongolia Autonomous Region. The objective was to validate the mediating role of psychological resilience in the impact of the sense of isolation among rural empty-nest elderly on their self-care ability in old age. The scores for feelings of alienation among rural elderly living alone were 45.52 ± 5.28. The scores for psychological resilience were 80.21 ± 6.35, and the scores for self-care capacity in old age were 140.45 ± 17.59. A negative correlation was observed between feelings of alienation and psychological resilience (*r* = −0.459, *P* < .001), as well as between feelings of alienation and self-care capacity (*r* = −0.651, *P* < .001). Conversely, a positive relationship was identified between psychological resilience and self-care capacity (*R* = 0.452, *P* < .001). Psychological resilience plays a mediating role in the impact of feelings of alienation on self-care capacity among the rural elderly living alone. The sense of alienation experienced by elderly individuals living alone in rural areas can directly impact their self-care ability in old age. Additionally, it can indirectly affect their self-care capacity by influencing their level of psychological resilience.

## 1. Introduction

With the continuous advancement of urbanization in China over the past 2 decades,^[1–3]^ rural areas have experienced significant population mobility, resulting in a growing number of elderly individuals facing severe aging care challenges in the absence of their children. Concurrently, national data indicates a persistent decline in China fertility rate, which dropped from 2.6 births per woman in the late 1980s to approximately 1.3 in recent years.^[4,5]^ This decline reduces the likelihood of elderly individuals having adult children available for caregiving, thus exacerbating the increase in the number of childless elderly and intensifying the “empty nest” phenomenon in rural areas. Consequently, aging care for rural empty-nesters has emerged as a focal point of societal concern.

In this study, we focus on “empty nest” elderly, typically those over 60 years old, who are either childless or unable to cohabit with their children long-term.^[6]^ Due to the limited availability of aging care facilities in rural areas and the long-term absence of familial support, traditional familial and institutional care approaches are insufficient to address their comprehensive needs.^[7]^ In this context, “self-care capabilities,” which encompass financial self-sufficiency, daily living skills, and health self-management,^[8]^ become particularly crucial. As the rural “empty nesting” phenomenon intensifies, an increasing number of elderly individuals are compelled to seek methods to enhance their self-care capabilities independently.^[9,10]^ Thus, this study significantly contributes to understanding and promoting self-care capacity among rural empty-nesters.

Additionally, we examine the impact of alienation on these individuals. Alienation refers to an individual’s subjective sense of disconnection from society, loss of personal identity, and diminished control over their external environment. The rapid advancement of social informatization, accompanied by limited digital infrastructure in rural regions, contributes significantly to this alienation by excluding rural elderly from digital engagement and social participation.^[11–13]^ Empirical research has consistently demonstrated that heightened alienation negatively affects psychological well-being and quality of life among older adults, thereby reducing their self-care capabilities.^[14,15]^

Moreover, we explore psychological resilience – defined as individuals’ ability to withstand adversity or stress.^[10]^ Enhanced psychological resilience is positively correlated with better quality of life and improved self-care capabilities among elderly populations. The novelty of this study lies in its examination of the mediating role of psychological resilience in the relationship between alienation and self-care capabilities, a relationship that previous research has inadequately explored.

Therefore, this study aims to investigate the interconnected relationships among alienation, psychological resilience, and self-care capabilities in rural empty-nesters. By proposing and testing the mediating role of psychological resilience, we aim to provide valuable insights and recommendations for formulating comprehensive self-care interventions for rural empty-nesters and to advocate for greater societal attention toward elderly care.

## 2. Subjects and methods

The study was ethically reviewed and approved by the Ethics Committee of Hulunbuir City People’s Hospital (Reference Number: CF2021017). Consent from the study participants was obtained verbally. In this study, questionnaires were distributed and collected on-site. The surveyors, consisting of trained researchers, explained the purpose and significance of the study to the participants prior to administering the questionnaires, ensuring informed consent was obtained. During the survey, if participants had any questions, the researchers provided explanations using a standardized set of instructions. Incomplete questionnaires were considered invalid.

### 2.1. Subjects

Between February 2022 and March 2023, employing purposive sampling alongside considerations of convenience, this study initiated its survey by first establishing contact and necessary communication with township and village leaders within the research area. These officials, guided by the inclusion criteria, assisted the researchers in recruiting elderly individuals living alone as subjects for the study. The inclusion criteria for this research were as follows: As of January 2022, individuals aged 60 years or older; Residents registered in rural areas and who have been living continuously in their residences for 6 months or more; Individuals either without children or those whose children do not live with them and visit less frequently than once a week; Individuals capable of engaging in normal communication and who could cooperate with the survey;

Participants who were informed about the study and voluntarily agreed to participate;

Individuals living alone or only with their spouse. Exclusion criteria included individuals: suffering from severe organic diseases, such as malignant tumors, serious cardiovascular diseases, etc; with past or current mental health issues; not living with their children but residing nearby enough to receive regular care from them; who were uncooperative during the survey or provided low-quality responses to the questionnaire.

In this study, a total of 1000 questionnaires were distributed, with 925 valid questionnaires retrieved, resulting in an effective response rate of 92.50%.

### 2.2. Measurement

#### 2.2.1. Self-constructed questionnaire

In the study, a self-constructed questionnaire was utilized to collect general information. The content of the questionnaire included demographic and lifestyle characteristics of rural elderly living alone, such as gender, age, educational background, monthly expenditures, smoking habits, alcohol consumption, marital status, and types of chronic diseases, among other relevant data.

#### 2.2.2. Alienation scale

The Alienation Scale, developed by YANG D in its Chinese version, was employed to assess the sense of alienation among rural elderly living alone.^[16]^ This scale encompasses 4 dimensions: social isolation, powerlessness, self-estrangement, and sense of meaninglessness, consisting of 15 items in total. Each item is scored on a scale from 1 to 4, with the total score ranging from 15 to 60. A higher total score indicates a more severe level of alienation in the rural elderly living alone. Sample items include “Sometimes I feel that the people I know are not very friendly” for social alienation and “Most of the things I do daily are very valuable and meaningful to me” for meaninglessness. The scale has been validated in Chinese populations, demonstrating good reliability with a Cronbach alpha of 0.881,^[16]^ indicating that the scale is also effective for use with Chinese samples. In our study, the overall Cronbach alpha for the SAS was 0.878, and the Cronbach α for each subscale ranged from 0.815 for powerlessness to 0.912 for feelings of self-alienation in this study.

#### 2.2.3. Psychological resilience scale

The Psychological Resilience Scale, developed in its Chinese version by Huang Weixiao, was used to evaluate the psychological resilience of rural elderly living alone.^[17]^ This scale includes 5 dimensions: calmness, confidence, comfort, perseverance, and the experience of a meaningful life, with a total of 25 items. Each item is scored between 1 and 7, with the overall score ranging from 25 to 175. Higher scores denote greater psychological resilience among the rural elderly living alone. Sample items include “I can get through difficult times because I’ve experienced difficulty before” for persistence and “When I make plans, I will follow through with them” for self-reliance. The Chinese version of the scale has demonstrated good reliability, with a Cronbach alpha of 0.943, and the subscale Cronbach alphas range from 0.897 to 0.903, confirming its robustness and validity in Chinese samples.

#### 2.2.4. Self-care capacity for elderly scale

The Self-Care Capacity for Elderly Scale, developed by Pang Shuqin in its Chinese version, was utilized to assess the self-care ability of rural elderly living alone.^[18]^ This scale is comprised of 3 dimensions: financial independence, daily living skills, and health self-maintenance, totaling 45 items. Each item is scored from 1 to 5, with the total score varying from 45 to 225. Higher scores indicate a stronger self-care ability among the rural elderly living alone. Sample items include “I have adapted to the current living environment (living pace, weather, etc)” for daily living skills and “I can maintain good interpersonal relationships” for health self-maintenance. In this study, the Cronbach α coefficient is 0.952, the Cronbach α for the Self-Care Ability Scale’s 3 dimensions were 0.876, 0.922, and 0.939, respectively. Additionally, a KMO coefficient of 0.936 underscores the scale’s good reliability. The distribution of subscale scores was checked to ensure they meet the assumptions required for regression analysis, and this information is included in the results section.

### 2.3. Statistical analysis

The analysis of the data was carried out using the SPSS 26.0 statistical software package. Qualitative data were represented by frequencies and proportions. Before performing Pearson correlation analysis, assumptions including normality and linearity were checked, and the data were confirmed to meet these assumptions. Therefore, Pearson correlation was appropriate to examine the relationships between alienation, psychological resilience, and self-care capability in aging. The Harman single-factor test was employed to assess common method bias. The mediating effect of psychological resilience between alienation and self-care capability in aging was analyzed using Model 4 of the Process 4.1 plugin, which was suitable for testing simple mediation aligned with our conceptual framework. The mediation effect was tested using the Bootstrap method with 5000 resamples to calculate the 95% confidence interval. A *P*-value of <0.05 was considered statistically significant.

## 3. Results

### 3.1. The demographic data of rural empty-nest elderly, characterized by their sense of alienation, psychological resilience, and the current state of self-sufficiency in elder care

The results of this study indicate that the age range of the participants was between 61 and 82 years, with an average age of 70.34 ± 8.86 years. Regarding gender distribution, there were 423 males (45.72%) and 502 females (54.28%). In terms of educational background, 268 participants (28.99%) had an education level of elementary school or below, 501 participants (54.12%) had completed junior high school, and 156 participants (16.89%) had attained a high school diploma or higher. As for marital status, 318 elderly individuals (34.29%) were without a spouse, while 607 (65.71%) had a spouse. Concerning chronic diseases, 228 participants (24.65%) had no chronic diseases, 309 (33.41%) had 1, 258 (27.89%) had 2, and 130 (14.05%) had 3 or more. The score for feelings of isolation among rural elderly living alone was 45.52 ± 5.28, the score for psychological resilience was 80.21 ± 6.35, and the score for self-reliance in elderly care was 140.45 ± 17.59.

### 3.2. The relationship between rural elderly’s feelings of alienation, psychological resilience, and self-care ability in old age

There is a negative correlation between alienation and psychological resilience (*r* = −0.837, *P* < .001), as well as between alienation and self-care ability in old age (*r* = −0.533, *P* < .001). Conversely, a positive correlation exists between psychological resilience and the ability for self-care in old age (*R* = 0.509, *P* < .001).

### 3.3. The mediating effect of psychological resilience between alienation and self-care ability in elderly individuals

Firstly, standardize each predictor variable and test for mediating effects using hierarchical regression analysis. In the first step, self-reliance in elderly care is used as the dependent variable, with alienation as the independent variable. In the second step, psychological resilience is treated as the dependent variable, with alienation again serving as the independent variable. In the third step, self-reliance in elderly care is again the dependent variable, with both alienation and psychological resilience entering the regression equation as independent variables. Hierarchical regression analysis indicates that alienation negatively predicts self-reliance in elderly care among rural empty-nesters (β = −0.654, *P* < .001) and negatively predicts their psychological resilience (β = −0.459, *P* < .001). When alienation and psychological resilience are separately predicted for self-reliance in elderly care, the predictive effects of both are significant. When predicting self-reliance in elderly care among rural empty-nesters simultaneously with alienation and psychological resilience, alienation negatively predicts self-reliance in elderly care (β = −0.542, *P* < .001), while psychological resilience positively predicts it (β = 0.455, *P* < .001). This indicates that psychological resilience plays a partial mediating role in the prediction of self-reliance in elderly care by alienation, as shown in Table 1.

**Table 1:** Analysis of the mediating role of psychological resilience in the impact of feelings of alienation on self-care ability in aging.

Model	Dependent variable	Independent variable	Unstandardized regression coefficient	S. E	*t*-value	*P*-value
Model 1	Self-care ability in aging	Feelings of alienation	−0.738	0.028	−34.987	.000
Model 2	Psychological resilience	Feelings of alienation	−0.535	0.043	−20.265	.000
Model 3	Self-care ability in aging	Psychological resilience	0.540	0.016	29.336	.000
		Feelings of alienation	−0.449	0.025	−24.392	.000

S.E = standard error.

By employing the exploratory factor analysis of the Harman single-factor test method for the common method bias test, the variance explained by the unrotated first factor is 19.72%, which is below the critical threshold of 40%, and there are more than 1 factors >1. This suggests that the study does not suffer from a severe common method bias.

This study, taking the alienation of rural empty-nesters as the independent variable, self-reliance in elderly care as the dependent variable, and psychological resilience as the mediating variable, uses PROCESS 4.1 for model verification in the mediation effect analysis and further tests with Bootstrap bias-corrected bootstrapping. The 95% confidence intervals for each mediating effect, as shown in Table 2, indicate that the 95% CI of the mediating effect of psychological resilience between alienation and self-reliance in elderly care does not include 0, thus confirming the mediating role of psychological resilience between alienation and self-reliance in elderly care.

**Table 2:** Testing the mediating role of psychological resilience in the relationship between feelings of alienation and self-care ability in aging.

Path	Effect Value	S.E	LLCI	ULCI
Direct effect	−0.615	0.025	−0.664	−0.565
Intermediary effect	−0.289	0.026	−0.448	−0.344
Total effect	−0.904	0.029	−0.954	−0.862

LLCI = lower limit of the confidence interval, S.E = standard error, ULCI = upper limit of the confidence interval.

## 4. Discussion

This study aimed to investigate the relationship between feelings of alienation, psychological resilience, and self-care capability among rural empty-nest elderly in Northeast China, and to examine the mediating role of psychological resilience. The findings reveal the significant psychosocial challenges faced by this population and provide empirical evidence for the development of targeted intervention strategies.

The findings of this study indicate that the score for feelings of alienation among rural empty-nest elderly was 45.52 ± 5.28. This level is higher than that reported by Zhang Haimiao et al.^[7]^ for empty-nest elderly in the city of Xi’an 42.19 ± 7.61 and also exceeds the scores reported by Lü Shijie et al.^[14]^ for urban empty-nest elderly in Tai’an, Shandong Province 39.79 ± 11.78. We posit that this discrepancy is primarily attributable to the study’s focus on a rural population, in contrast to the urban settings of the other studies. This urban-rural divide is underpinned by deep-seated structural and cultural factors. First, regarding the accessibility of community services and resources, urban areas typically boast more comprehensive elderly care centers, medical service stations, and recreational facilities, offering diverse forms of social support. In contrast, the public service infrastructure in rural regions is comparatively underdeveloped, with a significant deficit in professionalized elderly care services. Second, the transformation of family structures and social networks has had a more pronounced impact on rural areas. The mass exodus of young and middle-aged laborers to urban centers has eroded the traditionally close-knit “acquaintance society” of rural communities. Consequently, the elderly not only face the absence of their children but also lose crucial peer support networks. Third, the digital divide exacerbates the social isolation of rural elderly. In today’s highly digitized world, urban elderly have greater opportunities to stay connected via the internet, whereas their rural counterparts are often excluded due to a lack of equipment, digital literacy, and reliable internet access, which further intensifies their feelings of alienation.

In terms of psychological resilience, the score for rural empty-nest elderly in our study was 80.21 ± 6.35. This is higher than the findings of Jiang Zhaoquan et al.^[15]^ for a similar population 76.49 ± 5.23 but lower than the results from Zhang Shaobo et al for urban empty-nest elderly 86.50 ± 8.40.^[18,19]^ The discrepancy with the study by Jiang Zhaoquan et al may be explained by its unique temporal context, as their data was collected in 2020 during the initial outbreak of the COVID-19 pandemic. While necessary public health measures, the strict lockdown and quarantine policies had a profound psychological impact on elderly populations. Specifically, prolonged social isolation disrupted daily community activities and family gatherings, significantly heightening feelings of loneliness. Fear of the virus and uncertainty about the future, particularly concerning their own health, drained their psychological reserves. Furthermore, disruptions to medical services and news of illness or death among peers acted as significant psychological stressors, which likely contributed to a systemic decline in the psychological resilience of the elderly during that period. Our study was conducted when the pandemic situation was relatively stable, which may account for the higher resilience scores observed.

Regarding self-care capability, the score for rural empty-nest elderly in our study was 140.45 ± 17.59, which is lower than the findings of Wang Donghua et al for rural elderly in the Hunan region 167.68 ± 18.80.^[20]^ This difference may be linked to the geographical locations of the studies – our research was conducted in the Northeast, while the comparative study was in a southern region. Regional socioeconomic disparities are a key explanatory factor. As a traditional industrial base, Northeast China has faced challenges of economic transition and continuous population outflow, leading to comparatively limited local government investment in public health and elderly care. In contrast, southern provinces like Hunan exhibit greater economic vitality, with more abundant social and medical resources. National statistics confirm a clear gap between China northern and southern regions in terms of per capita GDP, quality of urbanization, and public service provision. This macro-level disparity in economic and social resources translates directly to micro-level differences in the access elderly individuals have to medical information, health services, and financial support, ultimately manifesting in lower self-care capability scores.

Our study found that feelings of alienation were significantly negatively correlated with self-care capability, a finding consistent with the results of Wang Donghua et al.. This implies that the more severe the feelings of alienation, the weaker the self-care capability. This phenomenon is particularly acute in the rural Northeast, where economic stagnation has driven a large portion of the younger generation to migrate for work. The absence of children or being childless, compounded by the physiological decline associated with aging, creates numerous daily life difficulties for the rural empty-nest elderly, thereby intensifying their feelings of alienation. This profound sense of alienation can lead them to feel that life is losing its meaning and to lack motivation for life exploration, thus diminishing their capacity and willingness for self-care.

Furthermore, feelings of alienation were significantly negatively correlated with the level of psychological resilience, aligning with the findings of Liu Jia et al.^[21]^ This suggests that higher levels of alienation correspond to lower levels of resilience. A plausible reason is the severe social disconnection resulting from the long-term absence of children and the loss of peers. Research has demonstrated that chronic social disconnection fosters feelings of helplessness and isolation, which adversely affects the mental health of rural empty-nest elderly and, in turn, erodes their psychological resilience.^[22]^ Protracted feelings of helplessness and poor mental health, compounded by psychological issues, inevitably and significantly impair the self-care capability of the elderly.^[23]^

Conversely, a significant positive correlation was observed between psychological resilience and self-care capability, which is consistent with the research of Wang Donghua et al.. This may be because elderly individuals with higher psychological resilience are more confident and better able to assess and utilize available resources and social support, enabling them to more effectively cope with challenges and maintain their physical and mental health.^[24]^ Additionally, psychological resilience can empower rural empty-nest elderly to leverage their strengths and potential to meet their diverse caregiving needs.

Mediation analysis revealed that psychological resilience plays a partial mediating role in the relationship between feelings of alienation and self-care capability.

This statistical result has significant practical implications. It illuminates an underlying mechanism through which alienation impairs self-care: alienation not only has a direct negative effect on self-care behaviors but also operates indirectly by first eroding an individual’s psychological resilience. The degradation of this resilience is a critical step leading to the decline or abandonment of self-care. In practice, this means that intervention strategies should not be limited to the difficult external goal of reducing alienation (e.g., reversing macro-level migration trends). Instead, a more feasible pathway is to target psychological resilience as a core intervention point. Resilience acts as a “psychological firewall” or a “buffer.” An elderly person with strong resilience can better regulate their emotions and maintain a positive outlook even when facing an objectively alienating environment, thereby buffering the negative impact of alienation on their self-care capability.^[25]^ Therefore, efforts to care for the rural elderly should prioritize programs designed to cultivate and strengthen their psychological resilience.

To enhance the self-care capability of rural empty-nest elderly, strengthening their psychological resilience is paramount.

Here, we draw upon the Strength Model of Self-Regulation proposed by renowned psychologist Roy Baumeister. This theory posits that willpower and self-control function like a muscle: they are a limited resource that can be depleted through use (a phenomenon known as “ego depletion”) but can also be strengthened through regular “exercise.” This model provides a solid theoretical foundation for our recommendations: Promote Regular Physical Activity: Consistent physical exercise is not merely a physiological activity but also a form of willpower training. Adhering to a routine of walking, practicing Tai Chi, or engaging in senior fitness programs requires overcoming inertia and physical discomfort. This process of self-challenge serves to “exercise” the muscle of self-regulation, thereby enhancing psychological resilience. Guide the Establishment of Achievable Life Goals: Encourage the elderly to set small, concrete goals based on their individual circumstances, such as learning a new recipe, reading for 30 minutes daily, or tending to a small garden plot. Achieving each goal represents a successful act of self-regulation, fostering a sense of accomplishment and building self-efficacy. This, in turn, equips them with greater psychological resilience to better manage life’s difficulties and setbacks.

## 5. Limitations and future prospects

While this study offers valuable insights, it is not without limitations, such as the cross-sectional study design, which precludes the establishment of causal relationships.

Future research should focus on experimental interventions to more precisely identify psychological methods that can effectively enhance the resilience and reduce the alienation of rural empty-nest elderly. Specifically, future studies could design and test the efficacy of several interventions:

Cognitive-behavioral therapy: Delivered in group or individual formats to help the elderly identify and challenge the automatic negative thoughts that contribute to alienation and helplessness, thereby restructuring their cognitive frameworks.

Group counseling and support groups: Organize regular meetings for elderly individuals with shared experiences to exchange feelings and coping strategies, rebuild social connections, and provide mutual emotional support to combat loneliness.

Community-based mutual aid programs: Implement initiatives like a “time bank,” where healthier, younger-old individuals can provide services for older, more frail peers. Such reciprocal helping can enhance their sense of social value and belonging.

Reminiscence therapy: Guide the elderly to recall and share positive life experiences and accomplishments, helping them to rediscover a sense of meaning and self-worth in their later years.

By evaluating these specific interventions through rigorous randomized controlled trials, we can generate more precise, evidence-based recommendations for improving the self-care capability and overall quality of life for the rural empty-nest elderly.

## 6. Conclusions

The sense of alienation experienced by elderly individuals living alone in rural areas can directly impact their self-care ability in old age. Additionally, it can indirectly affect their self-care capacity by influencing their level of psychological resilience.

## Acknowledgments

We value the involvement of each participant and the engagement of all study members.

## Author contributions

**Data curation:** Li-Hong Yang.

**Investigation:** Lingyun Qu.

**Writing – original draft:** Li-Hong Yang.

**Writing – review & editing:** Li-Hong Yang.
